# Resistance Training Improves Respiratory Muscle Strength and Immune Response in Older Adults: A Randomized Clinical Trial

**DOI:** 10.3390/jfmk11040434

**Published:** 2026-10-19

**Authors:** Naiara Nadia Moreira-Silva, Meiry Souza Moura-Maia, Anamei Silva-Reis, Katielle Rodrigues Silva-Cardoso, Elano Silva-Sousa, Matheus Carrijo-Nascimento, Gustavo Paixão-Santos, Raianne Veiga-Celestino, Rosa Helena Ramos Paula-Vieira, Gaspar R. Chiappa, Andre Luis Lacerda Bachi, Regiane Albertini, Eleni Papakonstantinou, Daiana Stolz, Rodolfo P. Vieira

**Affiliations:** 1 Laboratory of Pulmonary and Exercise Immunology, Evangelical University of Goias (UniEvangélica), Avenida Universitária Km 3,5, Anápolis 75083-515, GO, Brazilraianneveiga00@gmail.com (R.V.-C.);; 2 Faculty of Health Sciences, Universidad Autônoma de Chile, Providencia, Santiago 7500912, Chile; 3 Postgraduate Program in Health Science, Santo Amaro University (UNISA), São Paulo 04829-300, SP, Brazil; 4 Department of Otorhinolaryngology–Head and Neck Surgery, Federal University of Sao Paulo (UNIFESP), Rua Pedro de Toledo 947, São Paulo 04038-032, SP, Brazil; 5 Laboratory of Pulmonary and Exercise Immunology, Federal University of Sao Paulo (UNIFESP), Rua Talim 330, São José dos Campos 12231-280, SP, Brazil; 6 Clinic of Pneumology, Medical Center, University of Freiburg, 79106 Freiburg im Breisgau, Germany; 7 Brazilian Institute of Teaching and Research in Pulmonary and Exercise Immunology (IBEPIPE), Rua Taguatinga, Anápolis 75064-420, GO, Brazil

**Keywords:** respiratory muscle strength, resistance training, Klotho, inflammaging, exercise immunology

## Abstract

**Background**: Aging is associated with sarcopenia, chronic low-grade inflammation, impaired functional capacity, and reduced respiratory muscle strength. Resistance training is recognized as an effective intervention to attenuate these age-related alterations; however, its effects on respiratory function and anti-aging biomarkers such as Klotho remain incompletely understood. **Methods**: In this randomized controlled trial, 60 community-dwelling older adults (60–75 years) were randomly allocated to a control group (n = 30) or a resistance training group (n = 30). Participants in the intervention group completed a supervised 12-week resistance training program (three sessions/week). Assessments performed before and after the intervention included anthropometry, body composition, respiratory muscle strength (maximal inspiratory and expiratory pressures), handgrip strength, 1 min sit-to-stand test, oxygen saturation, plasma levels of IL-6, IL-10, TNF-α, Klotho, and hematological parameters. **Results**: The resistance training group presented significantly increased maximal inspiratory and expiratory pressures, handgrip strength, and 1 min sit-to-stand performance while attenuating exercise-induced oxygen desaturation. The intervention also reduced plasma IL-6 concentrations and markedly increased circulating IL-10 and Klotho levels, indicating a favorable anti-inflammatory and anti-aging response. No significant changes were observed in systemic hematological parameters, whereas anthropometric adaptations were modest. **Conclusions**: Twelve weeks of resistance training significantly improved respiratory muscle strength and systemic inflammatory status in older adults. The concomitant increase in circulating Klotho suggests that resistance training may promote biological mechanisms associated with healthy aging, supporting its implementation as a non-pharmacological strategy to preserve functional independence and respiratory health in the elderly.

## 1. Introduction

Aging is accompanied by progressive declines in skeletal muscle mass, muscle strength, and physical performance, which increase the risk of sarcopenia, frailty, disability, and loss of independence in older adults [1,2,3]. These age-related changes have important implications for healthy longevity, as preservation of muscle strength and physical function is increasingly recognized as a major component of healthy aging [4]. Aging is also characterized by a chronic low-grade inflammatory state, commonly referred to as inflammaging, which is associated with increased circulating concentrations of pro-inflammatory mediators, including interleukin-6 (IL-6) and tumor necrosis factor-alpha (TNF-α) [5]. Persistent elevation o0f these inflammatory mediators may contribute to muscle catabolism, sarcopenia, and functional decline [5].

The mechanisms underlying age-related muscle decline are multifactorial and include anabolic resistance, hormonal changes, chronic low-grade inflammation, mitochondrial dysfunction, and reduced physical activity [1,2,5]. Notably, muscle strength declines more rapidly than muscle mass, highlighting the importance of muscle quality and neuromuscular function in age-related functional impairment [3]. Low muscle strength is strongly associated with adverse outcomes, including falls, impaired mobility, poor quality of life, and mortality [4]. Therefore, preserving skeletal muscle health is a major goal in aging research and clinical practice. Interventions such as resistance training and adequate protein intake may help to attenuate muscle loss and improve muscle function in older adults [4,6,7].

Resistance training is a structured form of exercise in which skeletal muscles generate force against external resistance, and it represents a central strategy for preserving muscle strength, physical function, and independence during aging [4,6,8,9]. In older adults, progressive whole-body resistance training commonly incorporates exercises involving major upper- and lower-body muscle groups, such as knee extension and flexion, leg press or equivalent lower-limb movements, and upper-body pushing and pulling exercises [8,9]. Current evidence supports regular resistance training performed approximately two to three times per week, using multiple sets and progressively adjusted loads, with training intensity and volume individualized according to functional status and training experience [8,9]. Training adaptations may also be influenced by variables such as load, volume, frequency, and progression [9]. Accordingly, the present study employed a supervised, progressive whole-body resistance training program performed three times per week for 12 weeks, comprising seven upper- and lower-body exercises, four sets of 8–15 repetitions, and progressively adjusted loads corresponding to approximately 65–80% of one-repetition maximum. The complete exercise prescription and progression criteria are described in the Materials and Methods section.

Beyond its effects on neuromuscular function, resistance training may influence biological pathways associated with aging [10,11]. Among these, α-Klotho has emerged as a potentially relevant exercise-responsive protein [12,13,14,15]. Klotho is a transmembrane protein whose extracellular domain can be cleaved and released into the circulation as soluble α-Klotho, allowing endocrine and paracrine actions in multiple tissues [16]. Reduced Klotho expression has been associated with aging and age-related functional deterioration, whereas soluble α-Klotho has been implicated in the regulation of oxidative stress, inflammation, cellular homeostasis, and skeletal muscle integrity [16,17]. Experimental evidence suggests that Klotho may modulate oxidative and inflammatory signaling, including NF-κB-related pathways, and contribute to cellular defenses against oxidative stress [18,19]. Emerging clinical evidence indicates that exercise may increase circulating soluble α-Klotho, including randomized evidence demonstrating increased soluble α-Klotho following strength training in pre-frail older adults [17,20]. Therefore, evaluating α-Klotho together with inflammatory mediators may provide additional insight into the systemic biological adaptations accompanying resistance training in older adults.

Collectively, this evidence provides a rationale for investigating whether a structured resistance training program can influence respiratory and peripheral muscle function, functional capacity, and circulating inflammatory- and aging-related biomarkers in older adults.

## 2. Materials and Methods

The study protocol was approved by the Ethics Committee of the Evangelical University of Goiás (UniEVANGÉLICA; approval no. 6.095.284), and all procedures were conducted in accordance with the Declaration of Helsinki. The clinical trial was registered at ClinicalTrials.gov (NCT06682221—https://clinicaltrials.gov/study/NCT06682221; accessed 6 September 2026) on 11th March 2025. Verbal and written informed consent was obtained from all participants. This study followed the CONSORT guidelines for randomized controlled trials. CONSORT Checklist and Flow chart are available as Supplementary File S1 and Figure S1 respectively.

### 2.1. Population and Study Design

The sample size was determined based on the primary outcome of respiratory muscle strength, using G*Power software, version 3.1 (Heinrich Heine University Düsseldorf, Düsseldorf, Germany) [21], based on previously published data [22]. Assuming a two-sided significance level of 5% (α = 0.05), a statistical power of 80% (1 − β = 0.80), and an expected moderate-to-large standardized effect size (Cohen’s d = 0.74) for the difference between groups, the estimated sample size was 30 participants per group. From 80 community-dwelling older adults recruited through social media advertisements and by the Open University for Older Adults (UniAPI), a community outreach program of UniEVANGÉLICA, sixty were enrolled by meeting the inclusion and exclusion criteria. All participants provided written informed consent before enrollment. Participants were randomly allocated to either a control group (n = 30; 69.46 ± 6.42 years; 10 men, 20 women), which received no exercise intervention, or a training group (n = 30; 67.70 ± 6.08 years; 9 men, 21 women), which underwent a structured resistance training program. Participants were randomly allocated in a 1:1 ratio to the control group or resistance training group using a computer-generated simple randomization sequence. The allocation sequence was generated by a researcher who was not involved in participant recruitment or baseline assessments. Allocation concealment was maintained by restricting access to the computer file containing the randomization sequence; researchers responsible for participant recruitment and baseline assessments did not have access to this file and therefore could not know upcoming group assignments before allocation. Following enrollment and baseline assessment, participants were assigned to the control group or resistance training group according to the computer-generated allocation sequence. As inclusion criteria, men and women aged 60–75 years who were able to complete the study assessments and participate in the exercise program were eligible. Participants were required to attend at least 80% of the prescribed training sessions; otherwise, they were excluded from the final analysis. As exclusion criteria, individuals with neurological disorders that could interfere with study assessments or safely performing the exercise protocol were excluded. No dropouts were observed and all measurements represent the analysis of all volunteers. Participant retention and adherence were facilitated by several characteristics of the study setting and intervention. Participants were recruited through the Open University for Older Adults (UniAPI), a well-established community outreach program of UniEVANGÉLICA with strong engagement among older adults interested in health, educational, social, and physical activity initiatives. The university is readily accessible by public transportation, with a public transportation stop located directly in front of the campus. In addition, Brazilian adults aged ≥65 years are legally entitled to free urban and semi-urban public transportation, thereby reducing transportation-related barriers for eligible participants. Additional adherence strategies included direct supervision of all training sessions, individualized progression of exercise loads according to participant performance and tolerance, regular contact with the research team, and systematic monitoring of attendance. Participants were required to attend at least 80% of the scheduled training sessions to meet the predefined adherence criteria. Baseline physical activity and previous exercise experience were assessed through an investigator-administered anamnesis. Participants were asked whether they practiced physical activity and, when applicable, how long they had practiced it, how many times per week, and what type of activity they performed. Based on this assessment, all participants included in the study reported no participation in structured and/or regular physical activity during the preceding 6 months, and none reported engagement in resistance training during this period. A validated physical activity questionnaire, accelerometry, or other objective physical activity monitoring method was not used.

### 2.2. Study Outcomes

The primary outcome was the change in respiratory muscle strength following the 12-week intervention, as assessed by maximal inspiratory pressure (MIP) and maximal expiratory pressure (MEP).

Secondary outcomes included functional capacity assessed by the 1 min sit-to-stand test (1 min STS), peripheral muscle strength assessed by handgrip strength, and peripheral oxygen saturation (SpO_2_). Plasma concentrations of IL-6, IL-10, TNF-α, and α-Klotho were measured to characterize systemic inflammatory and aging-related responses. Hematological outcomes included total leukocytes, neutrophils, lymphocytes, monocytes, eosinophils, platelets, and hemoglobin. Body composition, anthropometric parameters, SARC-F score, and estimated skeletal muscle mass were additionally assessed to characterize participants’ nutritional, muscular, and sarcopenia-related profiles. All outcomes were evaluated at baseline and after 12 weeks, allowing assessment of within-group changes and between-group differences in response to resistance training.

### 2.3. Resistance Training Protocol

The resistance training program comprised seven exercises targeting major upper- and lower-body muscle groups: lat pulldown, machine chest press, lateral shoulder raise, biceps curl, triceps pushdown, leg extension, and leg curl. The leg extension and leg curl exercises specifically targeted the knee extensor and flexor muscle groups, respectively, which are particularly relevant to lower-limb function in older adults. Participants trained three times per week for 12 consecutive weeks, with each session lasting approximately 60 min.

Because of the number of exercise stations available, participants trained simultaneously in groups of 10 and were divided into two subgroups of 5 participants. One subgroup initiated each session with the lower-body exercises, whereas the other initiated the session with the upper-body exercises. The exercise sequence assigned to each subgroup was standardized and remained unchanged throughout the intervention and across all three weekly training sessions. Upper- and lower-body exercises were not alternated within the prescribed sequence. Thus, although the starting portion of the session differed between the two subgroups because of equipment availability, each participant followed the same predefined exercise sequence throughout the 12-week intervention.

The first week was specifically dedicated to familiarization with the exercise equipment, movement patterns, ranges of motion, and the correct execution of each exercise under direct supervision. Throughout the subsequent training sessions, exercise technique continued to be monitored by the research team, with verbal and practical corrections provided whenever necessary to ensure appropriate and safe movement execution.

Following familiarization, participants underwent one-repetition maximum (1RM) testing for each resistance exercise according to a previously validated protocol [23]. Briefly, participants first performed a specific warm-up using a submaximal load. Thereafter, the load was progressively increased across successive attempts until the participant was able to complete only one repetition with correct exercise technique and through the required range of motion. The highest load successfully lifted for one complete repetition was recorded as the 1RM for that exercise. Adequate recovery intervals were provided between successive attempts to minimize the influence of fatigue [23].

Training loads were initially prescribed at approximately 65% of each participant’s baseline 1RM. Participants performed four sets of 8–15 repetitions per exercise, with 1–2 min of recovery between sets and exercises. No separate standardized warm-up or cool-down protocol was performed. However, the first set of each exercise was performed using a lower external load than the subsequent working sets to facilitate preparation for the exercise. Load progression was individualized for each participant and each exercise. When the prescribed repetitions could be completed through the required range of motion with appropriate technique and movement control during two consecutive sessions, the external resistance for that specific exercise was increased by approximately 2–10%, according to individual performance and tolerance.

All training sessions were directly supervised. Ten participants trained simultaneously, divided into two subgroups of five participants. Each subgroup was directly supervised by one trained member of the research team, corresponding to a direct supervision ratio of approximately one supervisor to five participants. These supervisors were undergraduate students in physical education and/or physical therapy who had been specifically trained before the study to supervise the resistance training sessions. Their activities were, in turn, directly overseen by an experienced MSc or PhD student in physical education or physical therapy.

During each exercise, repetition cadence and movement control were visually monitored on an individualized basis, with particular attention to controlled concentric and eccentric phases and the avoidance of excessively rapid, uncontrolled, or compensatory movements. When inappropriate execution was observed, verbal instructions and practical corrections were provided as necessary. Movement velocity was not objectively quantified using an instrumented velocity monitoring device, and RPE and RIR were not systematically recorded. Thus, training intensity and individual tolerance were monitored through the initial 1RM-derived loads, prescribed repetition range, individualized load progression, direct supervision, and observation of exercise performance rather than through velocity-based or perceived-exertion measures [24].

Training progression was additionally guided by direct observation of muscular fatigue and the participant’s ability to maintain appropriate exercise technique. Observable signs of fatigue included progressive slowing of repetition cadence, increasing difficulty in completing the concentric phase, loss of movement control, reduced ability to complete the prescribed range of motion, appearance of compensatory movements, and inability to complete the prescribed repetitions while maintaining correct technique. These responses were monitored individually during each supervised session. When signs of excessive fatigue or deterioration in movement quality were observed, verbal and/or practical corrections were provided and, when necessary, the external load was adjusted or additional recovery was allowed before exercise was continued [9,11].

For physiological and safety monitoring, heart rate, arterial blood pressure, peak expiratory flow, and peripheral oxygen saturation (SpO_2_) were assessed immediately before and after each training session. Pre-exercise measurements were obtained after 10 min of seated rest, and post-exercise measurements were obtained after a further 10 min of seated recovery following completion of the training session. These variables were not continuously monitored during the resistance exercises. Table 1 summarizes the resistance training intervention.

### 2.4. Body Composition and Anthropometric Assessment

Body composition was assessed by bioelectrical impedance analysis (BIA) using an Omron^®^ digital bioelectrical impedance scale (Omron Healthcare Co., Ltd., Kyoto, Japan). Participants were instructed to avoid vigorous physical exercise for 24 h and alcohol consumption for 48 h before assessment, to refrain from large meals for at least 4 h, and to empty their bladders immediately before testing. Measurements were performed barefoot according to the manufacturer’s standardized procedures, with participants standing on the platform electrodes while holding the hand electrodes with their arms extended in front of the body. BIA-derived estimates of lean body mass and body fat were recorded [25,26].

Body weight (kg) was measured using the same device with participants barefoot and wearing light clothing. Height (m) was measured to the nearest 0.1 cm using a Sanny^®^ stadiometer (American Medical do Brasil Ltd.a., São Bernardo do Campo, SP, Brazil), with participants standing upright and the head positioned in the Frankfurt plane [25,26]. Body mass index (BMI) was calculated as body weight divided by height squared (kg/m^2^).

Waist, hip, and calf circumferences were measured using a nonelastic anthropometric tape according to standardized procedures [25,26]. Waist circumference was measured midway between the lowest palpable rib and the iliac crest, whereas hip circumference was measured at the maximum gluteal protuberance [25,26]. Calf circumference was measured at the maximum circumference of the calf with participants standing upright, muscles relaxed, and body weight evenly distributed between both lower limbs [25,26]. Waist-to-hip ratio (WHR) was calculated by dividing waist circumference by hip circumference [25,26].

All body composition and anthropometric measurements were performed by the same trained evaluator, who had more than 12 years of experience in physical assessment, to minimize interobserver variability and improve measurement reliability.

### 2.5. Sarcopenia Risk Analysis

Sarcopenia risk was screened using the SARC-F questionnaire, with scores ≥4 considered suggestive of sarcopenia. Skeletal muscle mass (SMM) was estimated using the anthropometric equation proposed by Lee et al. [27], and the skeletal muscle mass index (SMI) was calculated by normalizing SMM to height squared (kg/m^2^). These measures were used to characterize participants’ sarcopenia-related muscle status. In summary, screening for sarcopenia was performed using the SARC-F questionnaire, which consists of five items assessing muscle strength, the need for assistance with walking, the ability to rise from a chair, the ability to climb stairs, and a history of falls. Each item is scored from 0 to 2, resulting in a maximum total score of 10. Participants with a SARC-F score ≥4 were classified as being at risk of sarcopenia [28,29]. In summary, skeletal muscle mass (SMM) was estimated using the anthropometric equation proposed by Lee et al. (2000) [27], while the skeletal muscle mass index (SMI) was calculated by normalizing the estimated SMM to height squared (SMM/height^2^, kg/m^2^) [28,29].

### 2.6. Respiratory Muscle Strength Assessment

Maximal inspiratory (MIP) and expiratory pressures (MEP) were assessed by manovacuometry to evaluate respiratory muscle strength [11,14,25,26]. Participants remained seated upright, with feet supported and a nasal clip in place. MIP was measured following expiration to near residual volume and a maximal sustained inspiratory effort against an occluded airway, whereas MEP was measured following inspiration to near total lung capacity and a maximal sustained expiratory effort. Three attempts were performed for each maneuver with standardized verbal encouragement, and the highest value was used for analysis. Results were expressed in cmH_2_O [11,14,25,26].

### 2.7. Lower Body Strength Assessment and Peripheral Oxygen Saturation (SpO_2_)

Functional exercise capacity and lower-limb muscular endurance were assessed using the 1 min sit-to-stand test (1 min STS), according to established procedures and reference values [11,14,25,26]. Participants were instructed to repeatedly stand up completely from and sit down on a standard chair as many times as possible within 1 min. The total number of correctly completed repetitions was recorded, with higher values indicating better functional performance. The 1 min STS is a simple, reliable, and valid functional test that has been widely used to assess exercise capacity across adult and older populations [14,25,26].

Peripheral oxygen saturation (SpO_2_) was measured by pulse oximetry before and after the 1 min STS test. The pre-test measurement was obtained after a 10 min seated resting period, whereas the post-test measurement was obtained following completion of the test. The change in SpO_2_ (ΔSpO_2_) was calculated as the difference between the post-test and pre-test values and was interpreted specifically as the SpO_2_ response associated with the 1 min STS test rather than as a generalized measure of exercise-induced oxygen desaturation [11,14,25,26].

### 2.8. Blood Collection and Biomarker Assessment

Venous blood samples (5 mL) were collected into sterile K3-EDTA tubes. Immediately after collection, a 25 µL aliquot of whole blood was subjected to automated hematological analysis using a Sysmex XN-800i hematology analyser (Sysmex Europe GmbH, Norderstedt, Germany) to determine hematological parameters, including leukocyte and platelet counts and leukocyte differentials [11,14,25,26].

The remaining blood was centrifuged at 1000× *g* for 7 min at 4 °C. Plasma was aliquoted and stored at −86 °C until analysis [11,12,14,25,26]. Plasma concentrations of soluble α-Klotho, interleukin-6 (IL-6), interleukin-10 (IL-10), and tumor necrosis factor-alpha (TNF-α) were determined using commercially available DuoSet^®^ enzyme-linked immunosorbent assay (ELISA) kits (R&D Systems, Minneapolis, MN, USA), according to the manufacturer’s instructions. The assay ranges were 78.1–5000 pg/mL for α-Klotho (DY5334-05), 9.4–600 pg/mL for IL-6 (DY206), 31.2–2000 pg/mL for IL-10 (DY217B), and 15.6–1000 pg/mL for TNF-α (DY210). Optical density was measured using a SpectraMax i3 microplate reader (Molecular Devices, San Jose, CA, USA) [11,12,14,25,26].

Assay performance was evaluated using the limits of detection (LOD) and intra- and inter-assay coefficients of variation (CV). The analytical characteristics were as follows: IL-6, LOD 0.70 pg/mL, intra-assay CV <6%, and inter-assay CV <8%; IL-10, LOD 3.9 pg/mL, intra-assay CV <6%, and inter-assay CV <8%; TNF-α, LOD 1.6 pg/mL, intra-assay CV <5%, and inter-assay CV <7%; and soluble α-Klotho, LOD 6.2 pg/mL, intra-assay CV <5%, and inter-assay CV <9% [11,12,14,25,26].

### 2.9. Statistical Analysis

All statistical analyses were performed using IBM SPSS Statistics (version 27; IBM Corp., Armonk, NY, USA), and graphical representations were generated using GraphPad Prism (version 8.0; GraphPad Software, San Diego, CA, USA). Data distribution was assessed using the Shapiro–Wilk test and visual inspection of distributions, and homogeneity of variance was evaluated using Levene’s test. Continuous variables are presented as mean ± standard deviation (SD), with mean differences and 95% confidence intervals (CIs) reported where appropriate. Intervention effects were primarily assessed using a two-way mixed repeated-measures analysis of variance (ANOVA), with group (control vs. resistance training) as the between-subject factor and time (pre vs. post) as the within-subject factor. The group × time interaction was considered the primary inferential test of the intervention effect, indicating whether the magnitude of change over the 12-week intervention differed between groups. When significant effects or interactions were identified, post hoc pairwise comparisons were performed with adjustment for multiple comparisons using the Holm–Bonferroni procedure. Between-group comparisons of change scores, effect sizes, and corresponding 95% CIs were used as complementary measures of the magnitude and precision of the intervention effect. Within-group pre–post comparisons were considered secondary analyses and were not interpreted independently as evidence of an intervention effect. Accordingly, a statistically significant pre–post change within one group accompanied by a non-significant change within the other group was not considered evidence of a differential treatment effect in the absence of a significant group × time interaction or between-group difference in change. Because baseline differences were identified for selected outcomes, particularly 1 min sit-to-stand performance, baseline-adjusted analyses were additionally performed as sensitivity analyses. The post-intervention value was entered as the dependent variable, group as the fixed factor, and the corresponding baseline value as a covariate. Before interpreting the conventional analysis of covariance (ANCOVA), the homogeneity-of-regression-slopes assumption was evaluated by testing the baseline × group interaction. When this interaction was non-significant, the conventional ANCOVA was interpreted and the adjusted group effect was reported. When the baseline × group interaction was significant, indicating non-parallel regression slopes, the conventional ANCOVA assuming a common baseline–outcome relationship across groups was not interpreted as a single adjusted treatment effect. These baseline-adjusted analyses were considered sensitivity analyses, whereas the prespecified group × time interaction remained the primary longitudinal test of the intervention effect. Effect sizes for ANOVA effects were expressed as partial eta squared (η^2^p), with values of approximately 0.01, 0.06, and 0.14 interpreted as small, moderate, and large, respectively. Cohen’s d was calculated for relevant pairwise and between-group change-score comparisons and interpreted as small (0.20), moderate (0.50), and large (0.80). Absolute changes (Δ = post − pre) were additionally calculated and compared between groups, with corresponding effect sizes and 95% CIs reported where appropriate. For variables showing substantial departures from parametric assumptions, appropriate transformations or non-parametric sensitivity analyses were performed. All statistical tests were two-tailed, and statistical significance was set at *p* < 0.05.

## 3. Results

### 3.1. Baseline Characteristics of the Volunteers

Baseline demographic, anthropometric, cardiovascular, functional, and body-composition characteristics of the control and resistance training groups are presented in Table 2, whereas pre–post changes and within-group comparisons are presented in Table 3. The groups were comparable for most variables at baseline; however, diastolic blood pressure was significantly higher in the control group (*p* = 0.006), and sit-to-stand performance was also higher in the control group (*p* = 0.032). Despite the baseline difference in diastolic blood pressure, no significant group × time interaction was observed for DBP [F(1,58) = 0.32, *p* = 0.573, η^2^p = 0.005]. Similarly, no significant group × time interactions were observed for SBP [F(1,58) = 0.22, *p* = 0.640, η^2^p = 0.004], resting HR [F(1,58) = 0.69, *p* = 0.411, η^2^p = 0.012], or double product [F(1,56) = 0.013, *p* = 0.909, η^2^p < 0.001] (Table 2), indicating no differential effect of resistance training on these resting cardiovascular outcomes.

### 3.2. Effects of Resistance Training on Anthropometric and Body Composition Measurements

Anthropometric and body-composition responses to the 12-week intervention are presented in Table 3. Overall, these outcomes remained relatively stable in both groups, and no significant group × time interactions were observed. Specifically, the interactions were not significant for body weight [F(1,58) = 0.62, *p* = 0.435, η^2^p = 0.011], BMI [F(1,50) = 1.23, *p* = 0.273, η^2^p = 0.024], fat-free mass [F(1,51) = 0.055, *p* = 0.815, η^2^p = 0.001], fat mass [F(1,49) = 0.68, *p* = 0.414, η^2^p = 0.014], skeletal muscle mass [F(1,51) = 2.13, *p* = 0.150, η^2^p = 0.040], and skeletal muscle index [F(1,58) = 1.83, *p* = 0.181, η^2^p = 0.031]. Likewise, no significant differential changes were observed for waist circumference [F(1,46) = 3.74, *p* = 0.059, η^2^p = 0.075], hip circumference [F(1,47) = 0.43, *p* = 0.513, η^2^p = 0.009], waist-to-hip ratio [F(1,58) = 0.043, *p* = 0.837, η^2^p = 0.001], right calf circumference [F(1,58) < 0.001, *p* = 0.992, η^2^p < 0.001], or left calf circumference [F(1,58) = 0.006, *p* = 0.938, η^2^p < 0.001]. Although some within-group changes were observed, these secondary comparisons did not correspond to significant group × time interactions (Table 2). Collectively, these findings indicate that resistance training did not significantly modify the assessed anthropometric or body-composition outcomes compared with the control condition.

### 3.3. Effects of Resistance Training on Respiratory Muscles and Functional Capacity

**Figure 1:**
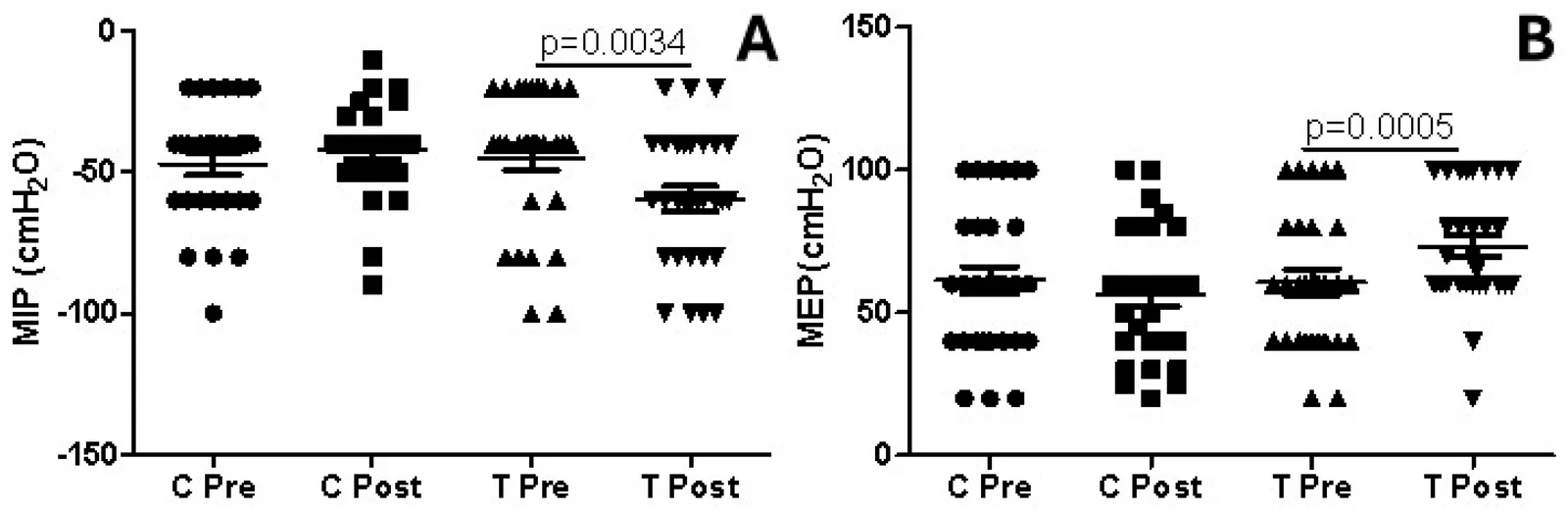
Resistance training group presents improved respiratory muscle strength in older adults. Maximal inspiratory pressure (MIP; (**A**)) and maximal expiratory pressure (MEP; (**B**)) were assessed before (pre) and after (post) the 12-week intervention in the control (C) and resistance training (T) groups. Significant group × time interactions were observed for both MIP and MEP. The *p*-values displayed in the figure represent secondary within-group pre–post comparisons, adjusted using the Holm–Bonferroni procedure where applicable. Data are presented as individual values with mean ± SD. C, control group; T, resistance training group; MIP, maximal inspiratory pressure; MEP, maximal expiratory pressure.

**Figure 2:**
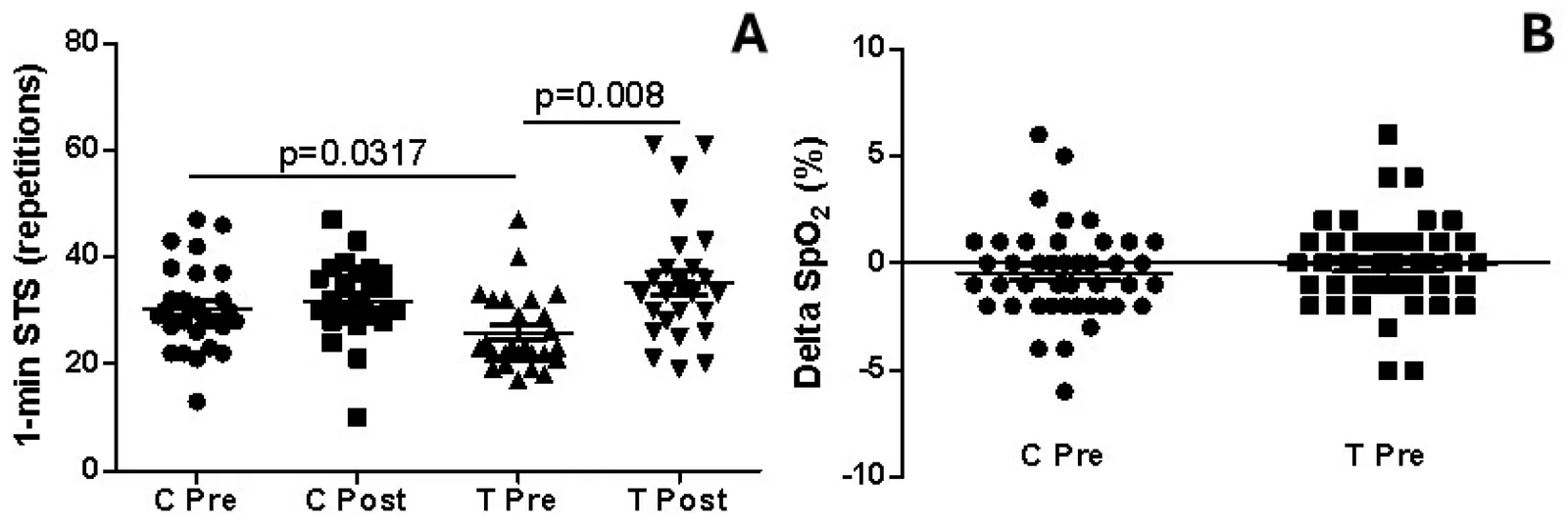
Resistance training group presented slight improvement in functional capacity among older adults. The 1 min sit-to-stand test (STS; (**A**)) and peripheral oxygen saturation (ΔSpO_2_; (**B**)) were assessed in the control (C) and resistance training (T) groups. A significant group × time interaction was observed for STS, whereas no significant between-group difference was observed for ΔSpO_2_. The *p*-values displayed in the figure represent the statistical comparisons indicated by the corresponding brackets and were adjusted using the Holm–Bonferroni procedure where applicable. Data are presented as individual values with mean ± SD. C, control group; T, resistance training group; STS, sit-to-stand test; SpO_2_, peripheral oxygen saturation; ΔSpO_2_, exercise-related change in peripheral oxygen saturation.

Respiratory muscle strength and functional capacity responses to the 12-week intervention are presented in Figure 1 and Figure 2, respectively. Significant group × time interactions were observed for MIP [F(1,58) = 10.95, *p* = 0.002, η^2^p = 0.159] and MEP [F(1,58) = 7.10, *p* = 0.010, η^2^p = 0.109], demonstrating greater improvements in inspiratory and expiratory muscle strength in the resistance training group than in the control group. Consistent with these interactions, secondary within-group comparisons showed significant pre–post improvements in the resistance training group for MIP (*p* = 0.0034; Figure 1A) and MEP (*p* = 0.0014; Figure 1B).

Functional performance also improved following resistance training (Figure 2A). A significant group × time interaction was observed for the 1 min sit-to-stand test [F(1,52) = 7.62, *p* = 0.008, η^2^p = 0.128], with a between-group difference in the change of 8.03 repetitions (95% CI 2.19–13.86). Because baseline STS performance was lower in the resistance training group than in the control group (*p* = 0.032), a baseline-adjusted ANCOVA was performed as a sensitivity analysis. The homogeneity-of-regression-slopes assumption was satisfied (*p* = 0.828); however, the adjusted group effect did not reach statistical significance [F(1,51) = 3.21, *p* = 0.079, η^2^p = 0.059], with an adjusted between-group difference of 4.71 repetitions (95% CI −0.57 to 9.99). Thus, although the primary longitudinal analysis demonstrated a differential improvement in STS performance, this finding was attenuated after adjustment for the baseline imbalance.

Exercise-related results in peripheral oxygen saturation (ΔSpO_2_) are presented in Figure 2B. No significant difference in ΔSpO_2_ was observed between the resistance training and control groups (mean difference +0.43 percentage points, 95% CI −0.45 to 1.30; *p* = 0.335), providing no evidence for a differential effect of resistance training on exercise-related oxygen desaturation.

### 3.4. Effects of Resistance Training on Circulating Inflammatory and Regulatory Biomarkers

**Figure 3:**
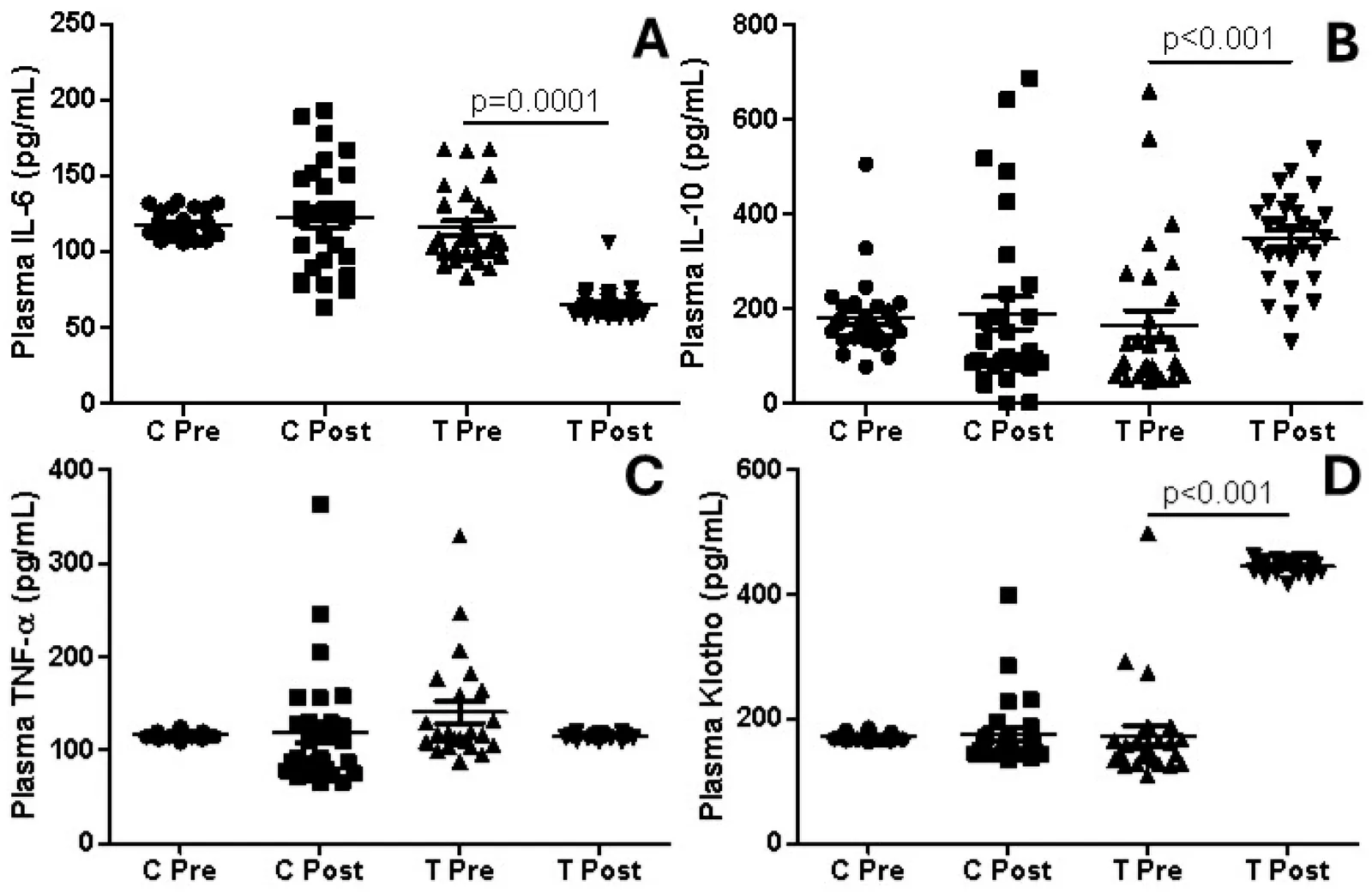
Resistance training group presented a modulation of pro- versus anti-inflammatory plasma cytokines and the anti-aging protein Klotho. Plasma concentrations of IL-6 (**A**), IL-10 (**B**), TNF-α (**C**), and α-Klotho (**D**) were assessed before (pre) and after (post) the 12-week intervention in the control (C) and resistance training (T) groups. Significant group × time interactions were observed for IL-6, IL-10, and α-Klotho, whereas the interaction for TNF-α was not significant. The *p*-values displayed in the figure represent secondary within-group pre–post comparisons and were adjusted using the Holm–Bonferroni procedure where applicable. Data are presented as individual values with mean ± SD. C, control group; T, resistance training group; IL, interleukin; TNF-α, tumor necrosis factor-alpha; SD, standard deviation.

Resistance training was associated with selective changes in circulating inflammatory and regulatory biomarkers (Figure 3). Significant group × time interactions were observed for IL-6 [F(1,56) = 7.82, *p* = 0.007, η^2^p = 0.123] and IL-10 [F(1,56) = 12.00, *p* = 0.001, η^2^p = 0.176], reflecting a reduction in IL-6 and an increase in IL-10 in the resistance training group. In contrast, the group × time interaction for TNF-α did not reach statistical significance (*p* = 0.057), and the between-group difference in change was also nonsignificant (−31.20 pg/mL, 95% CI −64.37 to 1.96; *p* = 0.065). Thus, although TNF-α decreased within the resistance training group, this secondary within-group finding should not be interpreted as evidence of a differential intervention effect.

Plasma α-Klotho also showed a significant group × time interaction [F(1,56) = 10.64, *p* = 0.002, η^2^p = 0.160], characterized by an increase following resistance training (Figure 3). Collectively, these findings indicate a differential biomarker response to the intervention, characterized by reduced IL-6 and increased IL-10 and α-Klotho, whereas a differential effect on TNF-α was not confirmed.

**Figure 4:**
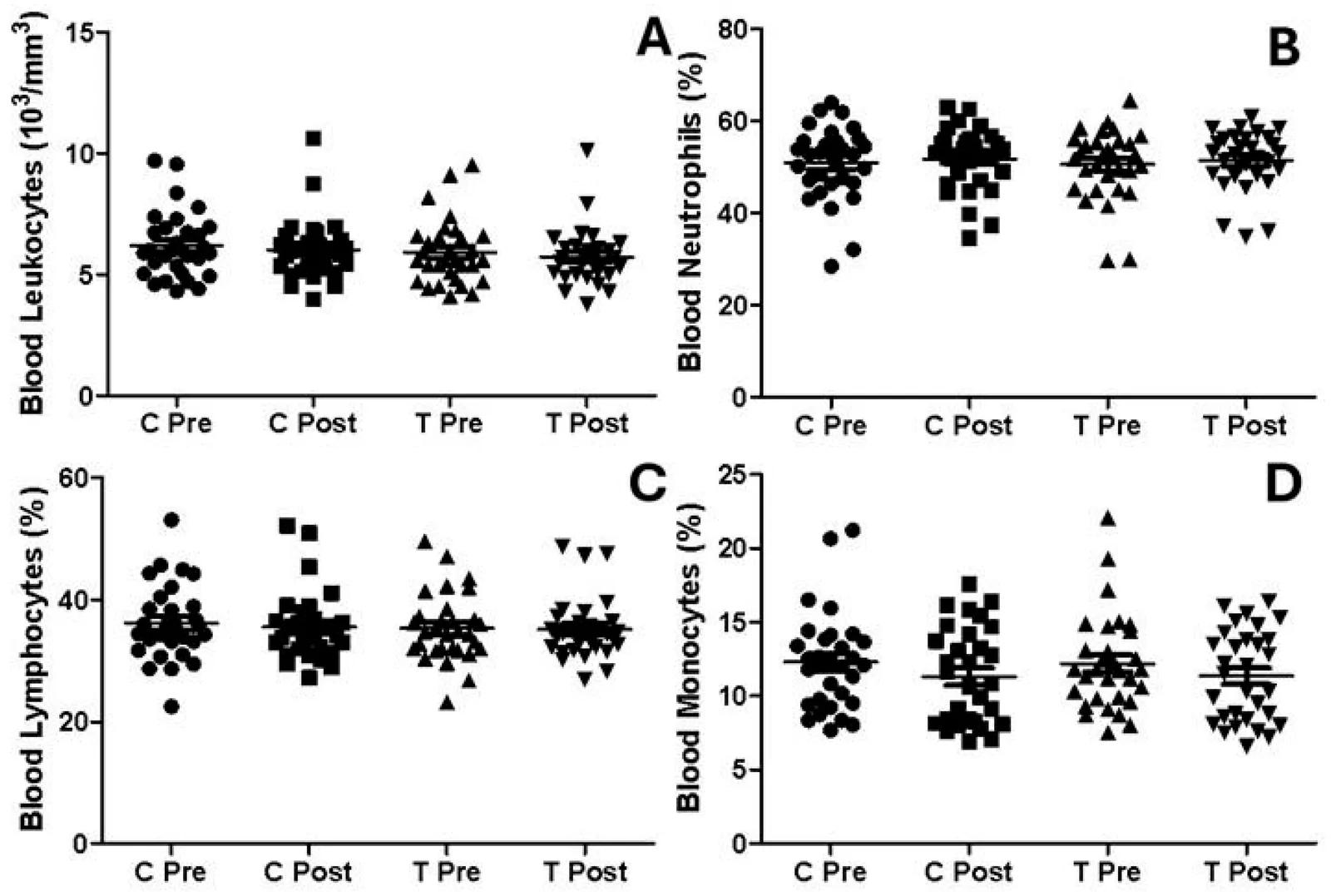

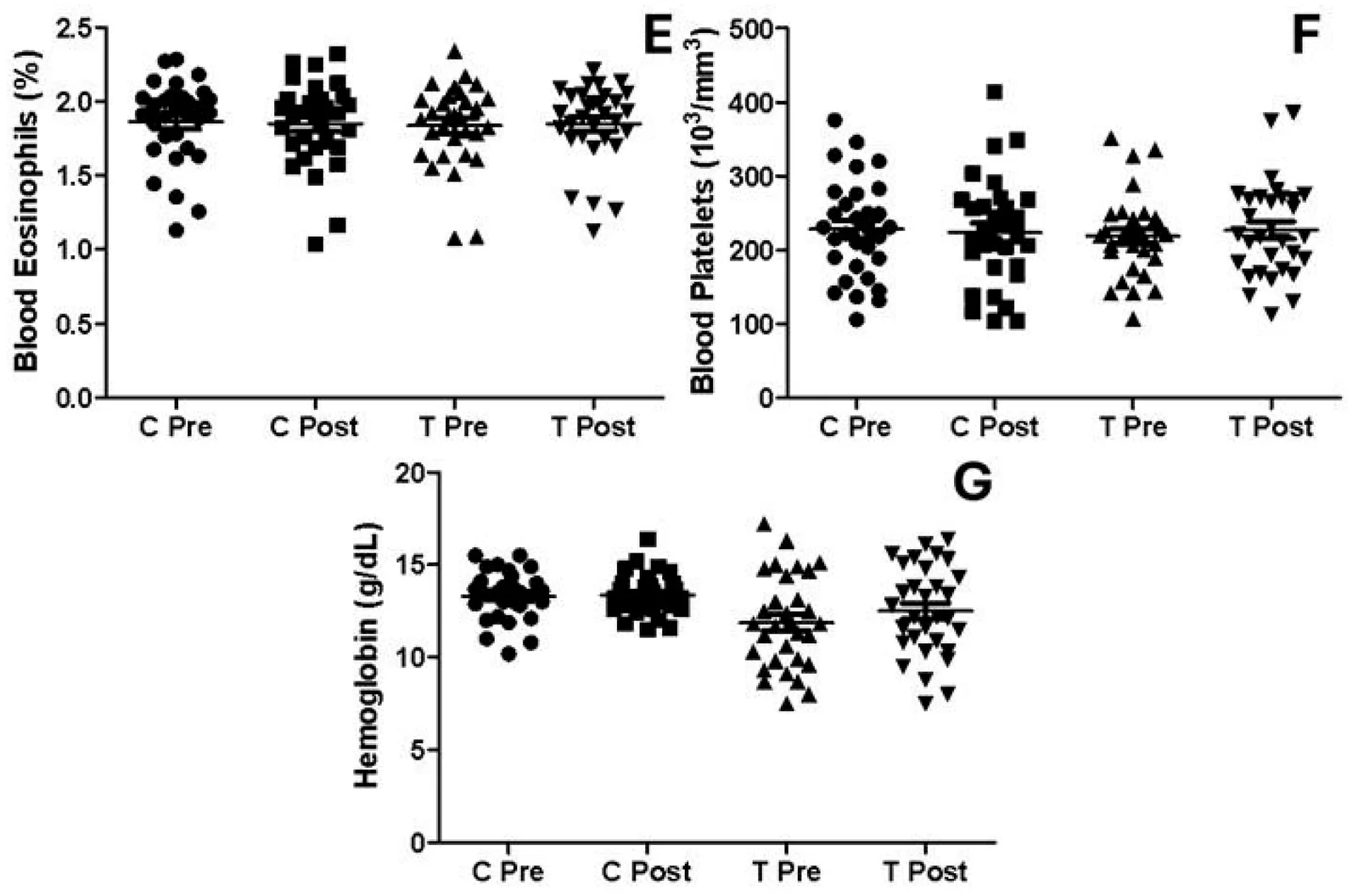
Hematological parameters remain unchanged in the resistance training group. Total leukocytes (**A**), neutrophils (**B**), lymphocytes (**C**), monocytes (**D**), eosinophils (**E**), platelets (**F**), and hemoglobin (**G**) were assessed before (pre) and after (post) the 12-week intervention in the control (C) and resistance training (T) groups. No significant differential changes were observed between groups over time for the assessed hematological parameters.

Hematological outcomes are presented in Figure 4. No significant changes were observed in total leukocytes, neutrophils, lymphocytes, monocytes, eosinophils, platelets, or hemoglobin, with no evidence of differential responses between the resistance training and control groups over the intervention period.

Taken together, Figure 3 and Figure 4 demonstrated that, in the resistance training group, a selective association of changes in plasma biomarkers, characterized by reduced IL-6, increased IL-10, and elevated α-Klotho concentrations, without significant changes in the hematological parameters, was observed.

## 4. Discussion

The present study demonstrated that 12 weeks of resistance training promoted relevant physiological adaptations in older adults, including increased maximal inspiratory (MIP) and expiratory pressure (MEP), handgrip strength, and 1 min sit-to-stand (1 min STS) performance. These functional adaptations were accompanied by reduced plasma IL-6 and increased IL-10 and α-Klotho, whereas hematological parameters remained stable. Collectively, these findings indicate that the resistance training group exhibited simultaneous improvements in respiratory and peripheral muscle function and favorably modulated biomarkers associated with inflammaging and healthy aging [3,5,6,8,11,26].

Aging is associated with chronic low-grade inflammation (inflammaging), which contributes to muscle catabolism, sarcopenia, frailty, and functional decline [5,10,11,13,15,16,17,28]. The reduction in IL-6 and increase in IL-10 observed in the resistance training group are consistent with a more favorable systemic inflammatory profile. Persistently elevated inflammatory mediators, including IL-6, have been associated with loss of muscle mass and strength and adverse health outcomes in older adults [26,28,29,30,31], while systematic reviews and meta-analyses indicate that resistance training can reduce circulating inflammatory markers in middle-aged and older adults [32,33]. Conversely, IL-10 is a major regulator of immune responses that limits excessive inflammation [34,35]. Exercise may promote an anti-inflammatory environment through several interacting mechanisms [36,37]. Contracting skeletal muscle functions as an endocrine organ and releases myokines, including IL-6, whose biological effects depend on the cellular source and physiological context. In contrast to chronically elevated resting IL-6 associated with inflammaging, the transient increase in muscle-derived IL-6 during exercise can stimulate anti-inflammatory mediators, including IL-10 and IL-1 receptor antagonist (IL-1ra), while contributing to the suppression of TNF-α production [36,37]. With repeated training, these acute responses may contribute to longer-term adaptations involving reduced basal pro-inflammatory signaling, improved metabolic regulation, and modulation of immune function. Accordingly, the lower IL-6 and higher IL-10 concentrations observed in the resistance training group after 12 weeks may reflect, at least in part, these exercise-induced regulatory adaptations. However, because muscle-derived cytokine release, immune-cell phenotypes, and intracellular inflammatory signaling pathways were not directly assessed, these mechanisms remain plausible explanations rather than mechanisms demonstrated by the present study. In contrast to the significant differential responses observed for IL-6, IL-10, and α-Klotho, TNF-α did not demonstrate a statistically significant group × time interaction. Although TNF-α decreased significantly within the resistance training group, the between-group difference in change was not statistically significant. Therefore, the present findings do not provide sufficient evidence that resistance training produced a differential reduction in TNF-α compared with the control condition. The within-group reduction should instead be regarded as a secondary finding that may warrant further investigation. This finding is consistent with the heterogeneous TNF-α response to resistance training reported in older adults, with some studies demonstrating reductions and others showing little or no change [32,33,38]. Such variability may reflect differences in training characteristics, including duration, frequency, intensity, and total training volume, as well as participant characteristics such as baseline inflammatory status, body composition, health status, age, and sex. Indeed, meta-analytic evidence suggests that reductions in TNF-α may be more apparent with greater training volumes, higher weekly frequencies, and/or longer interventions, although these moderating effects have not been consistently demonstrated across studies. Differences in baseline inflammation may also influence the magnitude of the response, as individuals with greater metabolic or inflammatory burden may have greater potential for improvement. Therefore, the absence of a significant group × time interaction for TNF-α in the present study may reflect the mediator-specific nature of the inflammatory response and variability in participant and training characteristics rather than an absence of broader immunomodulatory adaptations.

The marked increase in circulating α-Klotho represents another important finding. Klotho is associated with longevity and participates in the regulation of oxidative stress, inflammation, mineral metabolism, endothelial function, and tissue homeostasis [39,40,41]. Clinical evidence indicates that resistance training may increase circulating α-Klotho. In a recent randomized trial, Rippl et al. [42] investigated community-dwelling prefrail older adults undergoing 12 weeks of classical strength training, power training, or a non-exercise control condition. Classical strength training was performed twice weekly using multiple resistance exercises, with three sets of 8–12 repetitions at approximately 60–80% of 1RM. Notably, circulating soluble α-Klotho increased following classical strength training but not following power training or in the control condition [20]. These findings are consistent with the present results and are particularly relevant because both studies employed progressive resistance training over 12 weeks within a comparable relative-intensity range, although the present intervention was performed three times weekly using four sets of 8–15 repetitions. Klotho also has anti-inflammatory properties and may modulate NF-κB-related pathways [39]. Thus, the concomitant increase in α-Klotho and IL-10 and reduction in IL-6 may represent an integrated biological response for the resistance training group. However, because NF-κB signaling, oxidative stress, mitochondrial function, and skeletal muscle tissue were not directly assessed, causal relationships cannot be established.

A major finding was the improvement in MIP and MEP, indicating adaptations extending beyond peripheral skeletal muscle. Respiratory muscle strength declines with aging, in parallel with age-related changes in respiratory system mechanics and skeletal muscle function [43,44,45,46]. Although conventional resistance training does not specifically target the diaphragm and intercostal muscles, loaded multijoint exercise requires ventilatory and trunk-stabilizing activity. Improvements in neural activation, motor-unit recruitment, overall strength, and neuromuscular performance may therefore contribute to increased respiratory pressures [47,48,49,50]. Reduced systemic inflammation and increased α-Klotho may also provide a biological environment more favorable to muscle function, although this remains mechanistic speculation [20,41,42]. Clinically, preservation of respiratory muscle strength is relevant because greater respiratory muscle strength has been associated with a slower decline in mobility in older adults [51].

The resistance training group also presented improved 1 min STS performance and handgrip strength. The 1 min STS is a practical functional test with evidence supporting its reliability and validity in community-dwelling older adults [52,53], whereas handgrip strength is an established marker of overall muscular function and is associated with function and mortality in older populations [54,55,56]. These improvements are consistent with neuromuscular adaptations to resistance training, including enhanced muscle activation and neuromuscular performance [57,58,59]. They also agree with systematic reviews and randomized evidence demonstrating that progressive resistance training improves muscle strength and physical performance in older adults [57,58,59]. In fact, the resistance training group increased performance by approximately nine repetitions, whereas only a small change was observed in the control group, resulting in a significant group × time interaction and between-group difference in change. However, STS performance was lower in the resistance training group at baseline. To address this imbalance, we performed an additional baseline-adjusted ANCOVA as a sensitivity analysis. Although the adjusted estimate remained numerically higher in the resistance training group, the adjusted group difference did not reach statistical significance (*p* = 0.079; adjusted difference, 4.71 repetitions; 95% CI: −0.57 to 9.99). Therefore, the STS findings should be interpreted cautiously: the prespecified longitudinal analysis supports a differential improvement over time, but this finding was not independently confirmed by the baseline-adjusted sensitivity analysis.

The magnitude of the functional gains is particularly relevant because they occurred after a relatively short, 12-week intervention and despite modest anthropometric changes. This reinforces the concept that improvements in physical function during resistance training are not necessarily dependent on large changes in body size or gross anthropometric measures. Current recommendations identify progressive resistance exercise as a central component of exercise prescription for older adults [48], and randomized evidence confirms that resistance training programs of approximately 8–12 weeks’ duration can improve physical function in this population [57,58]. Accordingly, functional and neuromuscular adaptations may precede or exceed readily detectable changes in body composition.

Importantly, these functional and molecular adaptations occurred without significant alterations in leukocytes, differential leukocyte percentages, platelets, or hemoglobin. This stability suggests that the intervention induced selective changes in circulating inflammatory mediators rather than broad alterations in hematological cell populations. Taken together, the improvements in respiratory muscle strength, handgrip strength, functional capacity, and selected inflammatory and aging-related biomarkers indicate that the resistance training group exhibited adaptations across several physiological domains. However, these adaptations were not accompanied by significant differential changes in resting SBP or DBP, indicating that the cardiovascular response was not uniform across the outcomes assessed. Although resistance training has been shown to reduce resting blood pressure in older adults, the magnitude of this response is heterogeneous and appears to be influenced particularly by baseline blood pressure, with greater reductions generally observed in individuals with prehypertension or hypertension [60,61]. Thus, the absence of a significant blood pressure response in the present study should be interpreted within the characteristics of the study population and emphasizes that improvements in muscular, functional, and inflammatory outcomes do not necessarily occur in parallel with detectable changes in resting arterial blood pressure over a 12-week intervention.

Furthermore, resting cardiovascular variables remained relatively stable throughout the intervention, with no significant group × time interactions for SBP, DBP, resting HR, or double product. Although DBP differed between groups at baseline, this difference should not be interpreted as an effect of resistance training because it preceded the intervention and did not translate into a differential change over time. Moreover, DBP does not enter into the calculation of double product, which is derived from SBP and HR. Accordingly, the absence of significant differential changes in SBP and HR is consistent with the absence of a significant intervention effect on double product.

This study has several limitations that should be acknowledged. First, although the sample size was determined a priori based on the primary outcome of respiratory muscle strength, the relatively small sample and the 12-week intervention period may limit the precision and generalizability of the findings and may have reduced the ability to detect smaller between-group effects. This consideration is particularly relevant because adaptations to resistance training in older adults may vary according to baseline functional status, training characteristics, and individual responsiveness. Second, blinding of participants and exercise supervisors is inherently difficult in exercise-training trials and should therefore be considered when interpreting the findings. In addition, body composition was estimated using bioelectrical impedance and anthropometric methods rather than reference imaging techniques such as dual-energy X-ray absorptiometry, which may limit the sensitivity for detecting relatively small changes in skeletal muscle mass [62]. Finally, although the resistance training group presented significant changes in circulating IL-6, IL-10, and α-Klotho, these findings should be interpreted as systemic biomarker responses rather than evidence of the cellular mechanisms responsible for the observed functional adaptations. Intracellular signaling pathways, oxidative stress, mitochondrial function, and skeletal muscle tissue were not directly assessed; therefore, causal or mechanistic relationships between the biomarker and functional responses cannot be established [10,11,12,13,14,15,20]. Future randomized trials with larger and more diverse samples, longer follow-up periods, and direct tissue and molecular assessments are warranted to confirm the durability of these responses and clarify the biological mechanisms underlying the effects of resistance training in older adults. In addition, although the resistance training program targeted both upper- and lower-body muscle groups, it included a greater number of upper-body exercises and did not include a multijoint lower-body exercise such as the leg press; therefore, future studies should investigate whether a greater volume and variety of lower-limb exercises could produce additional adaptations in muscle mass and functional capacity in older adults. In addition, baseline STS performance differed between groups, and although the prespecified group × time analysis demonstrated a significant differential improvement, the corresponding baseline-adjusted sensitivity analysis did not reach statistical significance; therefore, the STS finding should be interpreted with appropriate caution. From a clinical perspective, maintaining functional capacity is fundamental for independence and healthy aging, while inflammation and frailty are closely interrelated in older adults [5,10,11,12,13,14,15,20]. From a practical perspective, the present findings support the use of structured and progressive resistance training to improve respiratory and peripheral muscle function in older adults. The intervention associated with these adaptations consisted of three supervised sessions per week for 12 weeks, comprising seven exercises targeting major upper- and lower-body muscle groups, with four sets of 8–15 repetitions per exercise. Training was initiated at approximately 65% of individual 1RM and progressively increased toward 80% of 1RM, with external loads individually adjusted according to performance, tolerance, and the ability to maintain appropriate exercise technique and movement control. These parameters provide a practical framework for implementing resistance training in older adults; however, they should not be interpreted as an optimal or universal prescription. Training frequency, exercise selection, volume, and intensity should be individualized according to health status, baseline functional capacity, previous training experience, tolerance, and specific functional goals [48,57]. Nevertheless, the mechanistic interpretation of the biomarker changes requires caution. Future studies with larger samples, longer follow-up, and different training modalities and intensities should determine the persistence of these responses and investigate whether changes in α-Klotho and inflammatory mediators directly contribute to functional improvements.

In conclusion, 12 weeks of structured, progressive resistance training produced concurrent improvements in respiratory muscle strength and slight peripheral muscle strength, while showing a favorable effect on STS performance in the primary longitudinal analysis, although the latter finding was attenuated after adjustment for the baseline between-group difference; effects were accompanied by favorable changes in selected circulating inflammatory and aging-related biomarkers, particularly IL-6, IL-10, and α-Klotho. These findings extend the recognized benefits of resistance training beyond conventional skeletal muscle outcomes by demonstrating simultaneous respiratory, functional, and systemic biological adaptations within a relatively short intervention period. Although these adaptations were not accompanied by significant differential changes in resting arterial blood pressure and were not uniform across all inflammatory mediators, the overall findings reinforce resistance training as a relevant non-pharmacological strategy for supporting multiple physiological domains associated with healthy aging. Importantly, the supervised and progressive nature of the intervention provides a feasible framework for resistance training programs aimed at preserving functional independence and physiological resilience in older adults. Further studies with larger and more diverse populations and longer follow-up are warranted to determine the durability and broader clinical significance of these adaptations.

## Figures and Tables

**Table 1:** Representative weekly structure and progression of the resistance training intervention.

Training Variable	Session 1	Session 2	Session 3
**Exercises**	Seven upper- and lower-body exercises	Same exercises	Same exercises
**Sets × repetitions**	4 × 8–15	4 × 8–15	4 × 8–15
**Initial load**	~65% baseline 1RM	Individually progressed	Individually progressed
**Rest between sets/exercises**	1–2 min	1–2 min	1–2 min
**Exercise sequence**	Standardized by subgroup	Same sequence	Same sequence
**Load progression**	2–10% when progression criterion was achieved *	Same criterion	Same criterion
**Direct supervision**	1 supervisor per 5 participants	1:5	1:5
**Pre/post monitoring**	HR, BP, PEF, SpO_2_	HR, BP, PEF, SpO_2_	HR, BP, PEF, SpO_2_

**Abbreviations:** 1RM, one-repetition maximum; HR, heart rate; BP, blood pressure; PEF, peak expiratory flow; SpO_2_, peripheral oxygen saturation. * External resistance for an individual exercise was increased by approximately 2–10% when the prescribed repetitions were successfully completed through the required range of motion with appropriate technique and movement control during two consecutive sessions. One subgroup of five participants initiated the session with lower-body exercises and the other with upper-body exercises because of equipment availability; the sequence assigned to each subgroup remained unchanged throughout the intervention.

**Table 2:** Baseline anthropometric and clinical characteristics of the volunteers.

	Control Pre	Training Pre	*p* Value	95% CI
**Age (years)**	69.47 ± 6.54	67.67 ± 6.18	0.5263	−1.49 to 5.09
**Weight (kg)**	75.14 ± 15.61	68.72 ± 10.82	0.0950	−0.53 to 13.36
**Height (cm)**	1.59 ± 0.09	1.57 ± 0.07	0.4287	−0.0252 to 0.0585
**BMI (kg/m^2^)**	29.68 ± 5.71	27.72 ± 3.53	0.1624	−0.8175 to 4.732
**Lean Mass (%)**	25.23 ± 5.38	25.85 ± 4.9	0.6663	−3.505 to 3.261
**Fat Mass (%)**	39.10 ± 10.77	37.27 ± 10.72	0.5533	−4.32 to 7.97
**Systolic Blood Pressure (mmHg)**	138.57 ± 21.62	131.57 ± 19.14	0.1895	−3.55 to 17.55
**Diastolic Blood Pressure (mmHg)**	84.43 ± 13.64	75.33 ± 11.12	0.0063	2.67 to 15.53
**Heart Rate (bpm)**	69.03 ± 11.89	67.8 ± 10.35	0.6698	−4.53 to 6.99
**Double Product**	9563.14 ± 2102.76	8964.79 ± 1825.86	0.2522	−437.60 to 1634.29
**Hip (cm)**	102.83 ± 10.46	102.32 ± 6.23	0.8395	−4.52 to 5.54
**Waist (cm)**	90.30 ± 11.11	88.45 ± 7.79	0.5214	−3.90 to 7.59
**Waist-to-Hip-Ratio**	0.882 ± 0.08	0.879 ± 0.08	0.9106	−0.039 to 0.044
**Hand Grip Strength (kg)**	23.43 ± 10.64	22.40 ± 6.17	0.5166	−2.11 to 4.18
**SARC-F**	2.93 ± 2.36	2.33 ± 2.78	0.3718	−0.73 to 1.93
**Skeletal Muscle Mass**	22.46 ± 6.13	20.36 ± 4.70	0.178	−0.99 to 5.2
**Skeletal Muscle Index**	8.78 ± 1.89	8.31 ± 1.40	0.282	−0.39 to 1.32
**Right Calf (cm)**	35.75 ± 3.87	34.98 ± 2.95	0.390	−1.01 to 2.55
**Left Calf (cm)**	35.73 ± 3.87	35.08 ± 3.20	0.4324	−1.19 to 2.48
**Sit-to-Stand Test (repetitions)**	30.43 ± 7.90	25.88 ± 7.17	0.0320	−0.41 to 8.68
**Resting SpO_2_ (%)**	95.97 ± 2.88	97.00 ± 1.15	0.083	−2.21 to 0. 14

Data are presented as mean ± SD. The *p*-values refer to between-group comparisons at baseline. Mean differences are presented as control − resistance training with corresponding 95% confidence intervals (CIs). SD, standard deviation; CI, confidence interval.

**Table 3:** Effects of resistance training on anthropometric and clinical characteristics of the volunteers.

	Control Pre	Control Post	*p* Value	95% CI	Training Pre	Training Post	*p* Value	95% CI
**Age (years)**	69.47 ± 6.54	N/A	N/A	N/A	67.67 ± 6.188	N/A	N/A	N/A
**Weight (kg)**	75.14 ± 15.61	75.26 ± 15.46	0.757	−0.67 to 0.91	68.72 ± 10.82	69.31 ± 10.87	−0.33	−0.67 to 1.50
**Height (m)**	1.59 ± 0.09	N/A	N/A	N/A	1.57 ± 0.07	N/A	N/A	N/A
**BMI (kg/m^2^)**	29.68 ± 5.71	29.78 ± 5.81	0.545	−0.24 to 0.45	27.72 ± 3.53	27.59 ± 3.51	0.184	−0.34 to 0.07
**Lean Mass (%)**	25.23 ± 5.38	26.07 ± 5.05	0.1473	−0.31 to 1.99	25.85 ± 4.90	26.87 ± 5.22	0.0486 *	0.007 to −2.04
**Fat Mass (%)**	39.10 ± 10.77	38.25 ± 11.58	0.249	−2.33 to 0.63	37.27 ± 10.72	35.53 ± 11.40	0.035 *	−3.34 to −0.14
**Systolic Blood Pressure (mmHg)**	138.57 ± 21.62	135.46 ± 20.73	0.485	−12.07 to 5.87	131.57 ± 19.14	131.47 ± 19.65	0.983	−9.59 to 9.39
**Diastolic Blood Pressure (mmHg)**	84.43 ± 13.64	83.7 ± 9.85	0.715	−4.8 to 3.34	75.33 ± 11.12	76.63 ± 10.65	0.666	−4.81 to 7.41
**Heart Rate (bpm)**	69.03 ± 11.89	70.90 ± 12.39	0.396	−2.57 to 6.30	67.80 ± 10.35	67.33 ± 10.86	0.797	−4.15 to 3.22
**Double Product**	9563.14 ± 2102.76	9533.69 ± 1824.57	0.936	−779.72 to 720.82	8964.79 ± 1825.86	8869.28 ± 2065.39	0.831	−1004.84 to 813.80
**Hip (cm)**	102.83 ± 10.46	102.67 ± 11.90	0.859	−1.92 to 1.62	102.32 ± 6.23	101.52 ± 5.75	0.036 *	−1.54 to −0.06
**Waist (cm)**	90.30 ± 11.11	90.74 ± 11.68	0.504	0.45 to 3.42	88.45 ± 7.79	87.19 ± 6.64	0.027 *	−2.37 to −0.15
**Waist-to-Hip-Ratio**	0.882 ± 0.080	0.888 ± 0.072	0.488	−0.013 to 0.026	0.879 ± 0.080	0.884 ± 0.079	0.480	−0.008 to 0.017
**Hand Grip Strength (kg)**	23.43 ± 10.64	22.60 ± 9.32	0.170	−2.03 to 0.37	22.40 ± 6.17	24.77 ± 6.18	0.001 *	1.32 to 3.41
**SARC-F**	2.93 ± 2.36	2.60 ± 2.44	0.105	−0.74 to 0.07	2.33 ± 2.78	1.13 ± 1.43	0.037 *	−2.32 to −0.08
**Skeletal Muscle Mass**	22.46 ± 6.13	22.10 ± 6.42	0.319	−1.09 to 0.37	20.36 ± 4.70	20.61 ± 4.72	0.030	0.03 to 0.47
**Skeletal Muscle Index**	8.78 ± 1.89	8.64 ± 1.99	0.310	−0.42 to 0.14	8.31 ± 1.40	8.36 ± 1.39	0.172	−0.02 to 0.12
**Right Calf (cm)**	35.75 ± 3.87	35.67 ± 3.85	0.792	−0.67 to 0.51	34.98 ± 2.95	34.90 ± 2.82	0.663	−0.45 to 0.29
**Left Calf (cm)**	35.73 ± 3.87	35.95 ± 3.94	0.413	−0.33 to 0.77	35.08 ± 3.20	35.28 ± 3.01	0.140	−0.07 to 0.47
**Sit-to-Stand Test (rep.)**	30.43 ± 7.90	31.79 ± 7.00	0.434	−2.15 to 4.86	25.88 ± 7.17	35.27 ± 11.39	0.0006 *	4.46 to 14.31
**Resting SpO_2_ (%)**	95.97 ± 2.88	95.48 ± 1.77	0.328	−1.48 to 0.51	97.00 ± 1.15	96.21 ± 1.37	0.006 *	−1.33 to 0.24

Data are presented as mean ± SD. The *p*-values shown in the table refer to within-group pre–post comparisons. The group × time interaction from the two-way mixed repeated-measures ANOVA was considered the primary test of the intervention effect and is reported in the Results text. Within-group comparisons were considered secondary analyses and were adjusted for multiple comparisons using the Holm–Bonferroni procedure, where applicable. CI, confidence interval; SD, standard deviation. * Represent a statistically significance.

## Data Availability

The data presented in this study are available on request from the corresponding author. The data are not publicly available due to privacy and ethical restrictions related to the protection of research participants and the conditions of the informed consent.

## Supplementary Materials

The following supporting information can be downloaded at https://www.mdpi.com/article/10.3390/jfmk11040434/s1, File S1: CONSORT Checklist; Figure S1: CONSORT 2025 Flow Diagram.
