# Supplementary material for: Resistance Training Improves Respiratory Muscle Strength and Immune Response in Older Adults: A Randomized Clinical Trial
Source: J Funct Morphol Kinesiol. 2026 Oct 19;11(4):434. doi: 10.3390/jfmk11040434 (PMC13692514; doi:10.3390/jfmk11040434)
Supplement: Supplementary file 1 [file jfmk-11-00434-s001.zip › jfmk-4580593-supplementary.pdf]

Supplementary Figure and Supplementary File

Supplementary Figure S1

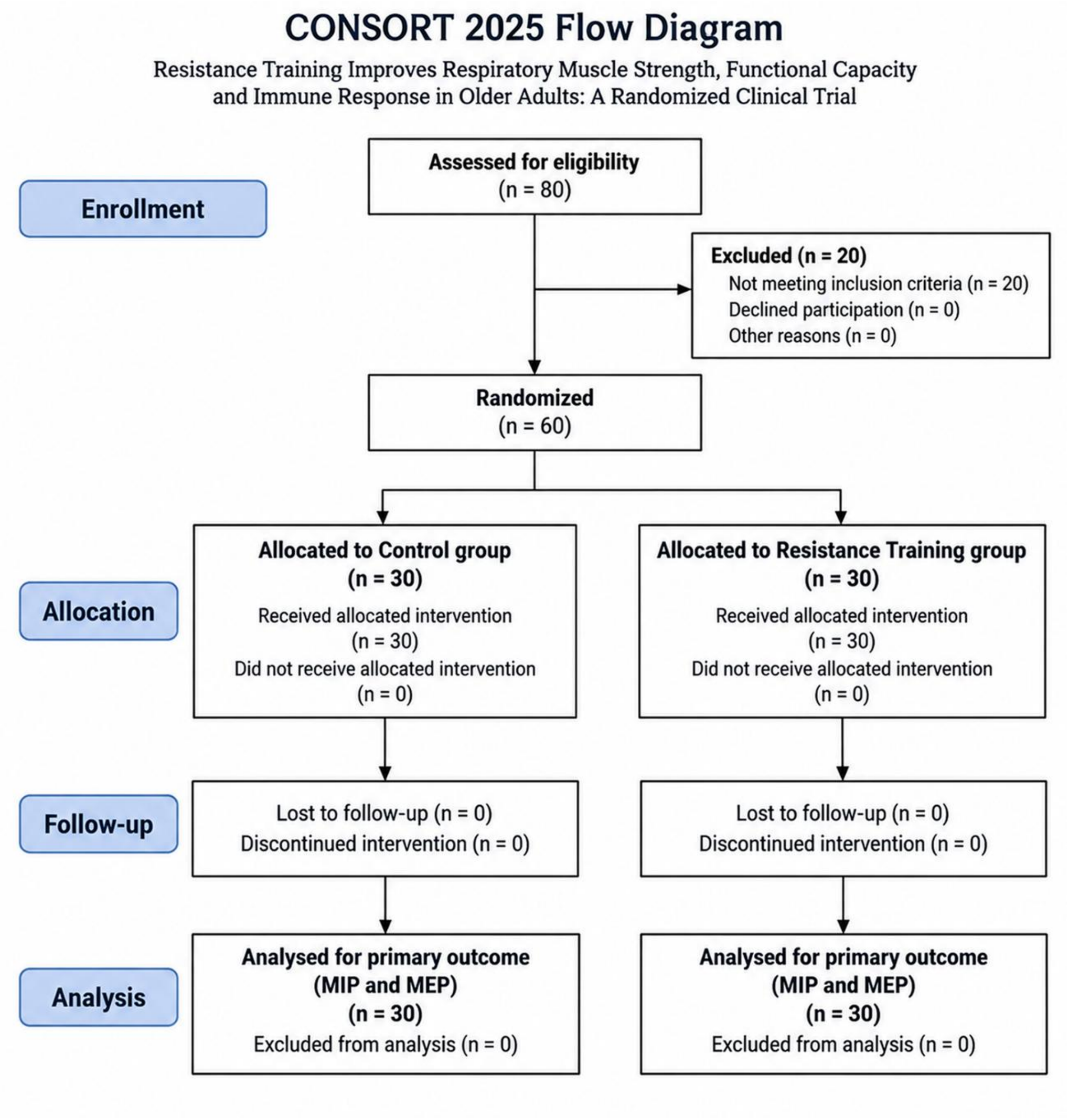

Supplementary File

Supplementary File S1 – CONSORT Checklist

CONSORT 2025 Checklist – Manuscript Assessment

Resistance Training Improves Respiratory Muscle Strength, Functional Capacity and Immune Response in Older Adults: A Randomized Clinical Trial

Section	Item	CONSORT 2025 requirement	Page(s)
Title and abstract	1a	Identification as a randomised trial	1
Title and abstract	1b	Structured summary of trial design, methods, results, and conclusions	2
Open science	2	Trial registry name, identifier/URL, and registration date	4
Open science	3	Where the trial protocol and statistical analysis plan can be accessed	10-11
Open science	4	Where/how de-identified participant data, data dictionary, statistical code, and other materials can be accessed	24
Open science	5a	Funding/other support and role of funders	24
Open science	5b	Financial and other conflicts of interest of authors	24
Introduction	6	Scientific background and rationale	3–4
Introduction	7	Specific objectives related to benefits and harms	4
Methods	8	Patient/public involvement in trial design, conduct, or reporting	N/A
Methods	9	Trial design, allocation ratio, and framework	5
Methods	10	Important post-commencement changes, including non-prespecified outcomes/analyses, with reasons	N/A
Methods	11	Trial settings and locations	5
Methods	12a	Eligibility criteria for participants	5
Methods	12b	Eligibility criteria for sites and intervention deliverers, if applicable	N/A
Methods	13	Intervention and comparator in sufficient detail for replication	5–7
Methods	14	Prespecified primary/secondary outcomes with measurement variable, analysis metric, aggregation, and time point	5–6
Methods	15	How harms/unintended effects were defined and assessed	N/A
Methods	16a	Sample-size determination and assumptions	5
Methods	16b	Interim analyses and stopping guidelines	N/A
Methods	17a	Who generated the random allocation sequence and method used	5
Methods	17b	Type of randomisation and restriction details	5
Methods	18	Allocation concealment mechanism	5

**Methods**	**19**	**Whether enrolling/assigning personnel had access to the allocation sequence**	**N/A**
**Methods**	**20a**	**Who was blinded after assignment**	**N/A**
**Methods**	**20b**	**How blinding was achieved and intervention similarity, if blinded**	**5**
**Methods**	**21a**	**Statistical methods for primary/secondary outcomes and harms**	**10–11**
**Methods**	**21b**	**Definition of who was included in each analysis and assigned analysis group**	**5**
**Methods**	**21c**	**Handling of missing data**	**N/A**
**Methods**	**21d**	**Methods for additional analyses, distinguishing prespecified from post hoc**	**11**
**Results**	**22a**	**Numbers randomised, receiving intended intervention, and analysed for primary outcome, by group**	**4–5 / Supplementary flow chart cited**
**Results**	**22b**	**Losses/exclusions after randomisation with reasons, by group**	**Supplementary flow chart cited**
**Results**	**23a**	**Recruitment and follow-up dates for benefits and harms**	**5**
**Results**	**23b**	**Why the trial ended/stopped, if relevant**	**N/A**
**Results**	**24a**	**Intervention/comparator as actually administered, including adherence and fidelity**	**5–7**
**Results**	**24b**	**Concomitant care received during the trial for each group**	**N/A**
**Results**	**25**	**Baseline demographic and clinical characteristics by group**	**36–37**
**Results**	**26**	**For each primary/secondary outcome: numbers analysed/available, group results, effect size and precision**	**12–18; 38–44**
**Results**	**27**	**All harms or unintended events in each group**	**N/A**
**Results**	**28**	**Other analyses, including subgroup/sensitivity analyses, distinguishing prespecified from post hoc**	**16–18 (limited)**
**Discussion**	**29**	**Interpretation consistent with results, balancing benefits/harms and other evidence**	**19–23**
**Discussion**	**30**	**Limitations: bias, imprecision, generalisability, and multiplicity where relevant**	**22–23**
